# Novel Heparan Sulfate-Binding Peptides for Blocking Herpesvirus Entry

**DOI:** 10.1371/journal.pone.0126239

**Published:** 2015-05-18

**Authors:** Pranay Dogra, Emily B. Martin, Angela Williams, Raphael L. Richardson, James S. Foster, Nicole Hackenback, Stephen J. Kennel, Tim E. Sparer, Jonathan S. Wall

**Affiliations:** 1 Department of Microbiology, The University of Tennessee, Knoxville, Tennessee, United States of America; 2 Department of Medicine, The University of Tennessee Graduate School of Medicine, Knoxville, Tennessee, United States of America; 3 Department of Radiology, The University of Tennessee Graduate School of Medicine, Knoxville, Tennessee, United States of America; 4 Department of Biomedical and Diagnostic Sciences, College of Veterinary Medicine, The University of Tennessee, Knoxville, Tennessee, United States of America; University of Regensburg, GERMANY

## Abstract

Human cytomegalovirus (HCMV) infection can lead to congenital hearing loss and mental retardation. Upon immune suppression, reactivation of latent HCMV or primary infection increases morbidity in cancer, transplantation, and late stage AIDS patients. Current treatments include nucleoside analogues, which have significant toxicities limiting their usefulness. In this study we screened a panel of synthetic heparin-binding peptides for their ability to prevent CMV infection *in vitro*. A peptide designated, p5+14 exhibited ~ 90% reduction in murine CMV (MCMV) infection. Because negatively charged, cell-surface heparan sulfate proteoglycans (HSPGs), serve as the attachment receptor during the adsorption phase of the CMV infection cycle, we hypothesized that p5+14 effectively competes for CMV adsorption to the cell surface resulting in the reduction in infection. Positively charged Lys residues were required for peptide binding to cell-surface HSPGs and reducing viral infection. We show that this inhibition was not due to a direct neutralizing effect on the virus itself and that the peptide blocked adsorption of the virus. The peptide also inhibited infection of other herpesviruses: HCMV and herpes simplex virus 1 and 2 *in vitro*, demonstrating it has broad-spectrum antiviral activity. Therefore, this peptide may offer an adjunct therapy for the treatment of herpes viral infections and other viruses that use HSPGs for entry.

## Introduction

Human cytomegalovirus (HCMV) is a beta-herpesvirus with nearly 90% prevalence in the adult human population in developing countries [1]. Initial viral infection is generally asymptomatic in immune competent individuals. However, severe CMV disease occurs in individuals with a deficient immune system (*e*.*g*., transplant patients suppressed to avoid graft rejection, late stage AIDS patients, and the developing fetus). In immune deficient adults, HCMV can cause pneumonitis, multi-organ disease, and death [1–3]. Retinitis and blindness are also common in HCMV-infected, late-stage AIDS patients in the absence of highly active antiretroviral therapies [3]. *In utero* infection can cause neurological sequela in infants, including sensorineuronal hearing loss (SNHL) and mental retardation [1, 4].

Attempts to develop a vaccine for CMV infection are ongoing but have met with limited success [5, 6]. Current regimens to treat HCMV infection (*i*.*e*., ganciclovir, foscarnet, and cidofovir) target viral DNA synthesis [7] but can have detrimental side effects [8]. Furthermore the increased use of these drugs has led to HCMV drug-resistance to these therapies [9–12]. Due to these limitations, it is clinically important to develop new therapeutics against HCMV that are selective, less toxic, and circumvent resistance. One avenue for drug development is to target other aspects of the HCMV life cycle besides genome replication.

One of these potential targets is virus attachment to the cell. HCMV uses heparan sulfate (HS) for entry into cells and to initiate viral replication [13, 14]. Virtually all cells express HS glycosaminoglycans as long un-branched chains associated with protein cores in the form of cell surface heparan sulfate proteoglycans (HSPGs) [15]. Heparan sulfate and heparin are both linear glycosaminoglycans (GAGs) composed of alternating glucosamine and uronic acids that can be *N*-acetylated and *N*-sulfated [15–17]. Although both HS and heparin are highly sulfated, HS has fewer modifications, making heparin more electronegative than HS GAGs [16, 17]. This is an important distinction as heparin is often used as a surrogate for HS GAGs in spite of these differences.

HSPGs act as docking sites for growth factors [16, 18], parasites such as the malarial sporozoite [19], pathologic amyloid-related proteins [20], and many human and non-human pathogenic viruses including HCMV [13] and herpes simplex virus (HSV) [21]. The HCMV envelope glycoproteins glycoprotein B (gB) and the glycoprotein M/N (gM/gN) heterodimer complex are involved in virus adsorption via interaction with HSPG expressed on the cell surface [13]. The ability of HS to act as a binding site for numerous distinct viruses can be attributed to its diverse structure and variable negative-charge density [15, 22, 23]. Despite the critical role that HS has in HCMV infection, therapeutics targeting HS to treat CMV infections are lacking. This is likely due to its ubiquitous expression on mammalian cells and its important role in facilitating the biological activity of growth factors.

Recently, a panel of heparin reactive peptides has been shown to preferentially bind the HSPG GAGs associated with pathologic deposits containing amyloid fibrils, *in vitro* and *in vivo* [24, 25]. Of these peptides, a synthetic, 31 amino-acid, polybasic peptide with a +8 net positive charge, designated p5, was shown to bind amyloid in visceral organs, including the liver, spleen, heart, and kidneys [26]. Notably, this peptide does not bind to HS-related GAGs expressed in healthy (*i*.*e*., amyloid-free) organs and tissues. Specific reactivity with amyloid-associated HSPGs and not healthy tissues is likely due to the fact that the amyloid-associated tissues are hypersulfated and electrochemically similar to heparin [27, 28]. Based on these properties, we hypothesized that these peptides could block CMV entry.

In this study we screened a panel of synthetic, heparan sulfate reactive, p5-related peptides to identify novel inhibitors of CMV HS-mediated adsorption and subsequent infection. We explored the mechanism of action of the peptide and whether it could prevent other viruses that use HS for entry.

## Materials and Methods

### Peptide synthesis and purification

Peptides were purchased from Keck Laboratories as semi-pure preparations. Routine purification was performed by HPLC (1100 series; Agilent) using elution from a reverse-phase C3 matrix in a linear gradient of 0–50% acetonitrile in water with 0.05% trifluoroacetic acid. Peptide peaks were eluted from the column using a flow rate of 1 mL/min; 1 mL fractions were collected, peak fractions were pooled, and the mass was determined by mass spectrometry (MS) using a single quadropole MS (Applied Biosystems). If multiple peaks were observed, peptides were further purified by RP-HPLC and the mass of each confirmed by MS. In all cases, the purified peptides used in these studies appeared as single peaks during HPLC purification and as single bands following electrophoresis by using SDS-polyacrylamide gel electrophoresis. The purified peptides were lyophilized as 5 mg aliquots and re-suspended in phosphate-buffered saline (150 mM NaCl, pH7.2; PBS) before use. The re-suspended peptides were stored at 4°C until use.

### Cells and virus

Low passage-number cells (< 20) were used for all the experiments. Mouse embryonic fibroblast 10.1 (MEF 10.1 [29]) were cultured in DMEM (Lonza, Rockland, ME) supplemented with Fetal Clone III serum (FCIII) to a final concentration of 10% (Hyclone, Logan, UT), Pen/Strep (P/S) to a final concentration of 100 U/ml and L-glutamine (L-Gln) to a final concentration of 2 mM. Human foreskin fibroblast cells (HFF; obtained from ATCC) were cultured in DMEM (Lonza, Rockland, ME) supplemented with Fetal Bovine Serum (FBS) to a final concentration of 10% (Hyclone, Logan, UT), L-Gln to a final concentration of 2 mM, and sodium pyruvate to a final concentration of 1 mM. Human Aortic Endothelial Cells (HAEC) were cultured in EGM-2 Bullet Kit (Lonza, Rockland, ME) supplemented with FBS to a final concentration of 6%. Human retinal pigment epithelia (ARPE-19) cells were cultured in Dulbecco’s Modified Eagle’s Medium (DMEM):F12 medium (Lonza, Rockland, ME) supplemented with FBS to a final concentration of 10%. Human normal lung fibroblast (MRC-5) cells cultured in Minimal Essential Medium (MEM) (Lonza, Rockland, ME) supplemented FBS to a final concentration of 10% and L-Gln to a final concentration of 2 mM. These lines were a kind gift from Dr. Mike McVoy, VCU. African green monkey kidney epithelial (VERO; ATCC) cells were cultured in DMEM media supplemented with FBS to a final concentration of 10%, sodium pyruvate to a final concentration of 1 mM, HEPES buffer to a final concentration of 10 mM and P/S to a final concentration of 100 U/ml.

MCMV RM4503 [30], was cultured *in vitro* in MEF 10.1 cells. The virus stock was titered using plaque assay (described below) and stored at -80°C. Bacterial artificial chromosome generated HCMV TB40/E-mCherry [31, 32] and TB40/E-pp150-GFP [33, 34] were cultured *in vitro* on HFF cells. The virus stock was titered using a plaque assay and stored at -80°C. Low passage number HCMV (passaged 2–3 times) was used for all experiments. Herpes Simplex Virus (HSV-1 KOS and HSV-2 186 Syn^+^) were cultured *in vitro* on VERO cells. The virus stock was titered using a plaque assay and stored at -80°C.

### Plaque reduction assay

Peptides were screened for their ability to reduce viral infection using a plaque reduction assay. Cells were cultured in 12-well (VERO) or 24-well culture plates (MEF 10.1 and HFF). When cells reached ~80% confluence the media was removed and washed once with PBS before addition of peptide. As a control, cells were incubated with PBS alone. After a 30 min incubation with peptide in PBS, virus (~100 pfu/well for MCMV and ~30–40 pfu/well for HCMV and HSV) was added and incubated for another 90 min (HSV and HCMV) or 60 min (MCMV). Following virus incubation the peptide/virus mixture was removed and replaced with 0.75% carboxymethyl cellulose (Sigma Aldrich, St. Louis, MO) (CMC) + complete media (DMEM + P/S + L-Gln) for MCMV and HSV experiments or 0.5% agarose (Lonza, Rockland, ME) in complete media for HCMV experiments. The plates were incubated at 37° C in 5% CO_2_ for 4 days and when plaques began to develop, plates were stained with Coomassie stain (AMRESCO, Solon, Ohio). Due to the inability of HCMV to form distinct plaques on HAEC and ARPE-19 cells, infection in these cell types was measured by counting mCherry positive foci 14 days post infection. Plaques were counted manually using a dissection microscope. Data was analyzed using Prism 5.0 (GraphPad Software, La Jolla, CA). Data were expressed as percent infection (100 x (number of plaques after treatment/ the number of plaques in the PBS-treated wells)).

### Flow cytometric analysis of attached virus

HFF cells were grown in a 24 well dish and allowed to reach ~80% confluency. The cells were cooled to 4°C to prevent virus internalization before addition of peptide (100μM) and incubated for ½ h. Following the incubation, HCMV TB40/E pp150-GFP was added (MOI 10) at 4°C and incubated for 1h. Following the incubation, cells were removed from the wells using non-enzymatic cell stripper solution (Corning), fixed (with paraformaldehyde) and the data acquired using a BD FACS Calibur flowcytometer (BD Biosciences). The data was analyzed using FlowJo software (TreeStar).

### Heparin blockade of peptide-mediated plaque reduction

Peptide p5+14 (100 μM) was pre-incubated with heparin sodium salt (Acros Organics, NJ) at different concentrations for 1 h at 37°C. This heparin/peptide mix was added to the cells and incubated for 30 min at 37°C. Following the incubation, supernatant was aspirated and cells washed once with PBS to remove unbound/excess heparin or peptide. The cells were subsequently infected with ~100 pfu/well of MCMV. To test whether heparin treatment of cells interferes with virus infection, MEF 10.1 cells in a 24 well dish were pre-incubated with different concentrations of heparin for 1 hour and washed as described above. Following this pre-incubation, infection was initiated as described above. Finally to test the effect of heparin treatment on the infectivity of virus, MCMV was incubated with different concentrations of heparin for 1h before infecting cells. For all treatments, virus was removed 1h post infection and cells were overlaid with CMC. Plates were incubated for 4 days before staining and counting the plaques.

### Enzymatic treatment of cells

Heparinase I, Heparinase II, Heparinase III, and Chondroitinase ABC were purchased from Sigma Aldrich (St. Louis, MO). MEF 10.1 cells in culture were treated with heparinase in heparinase buffer (20 mM Tris-HCl, pH 7.5, 50 mM NaCl, 4 mM CaCl_2_, and 0.01% bovine serum albumin (BSA)) at a concentration of 1U/ml or chondroitinase re-suspended in chondroitinase buffer (50 mM Tris, pH 8.0, 60 mM sodium acetate and 0.02% BSA) at a concentration of 1 U/ml for 1 h at 37°C. As a control, cells were treated with enzyme buffer alone. Following incubation, the enzyme solution was removed and cells were washed with PBS to remove excess enzyme. Subsequently the cells were treated with peptide and infected with virus. Data was collected and analyzed as described above.

### Visualization of bound peptide

Coverslips with fixed MEF cells were prepared, washed in PBS, and blocked with 1% BSA/PBS for 5 min. Following a PBS wash, the nuclei were stained using Hoechst (Life Technologies Molecular Probes, Grand Island, NY) 1:100 in H_2_O for 30 min at 37°C. Cells were then blocked using a casein block solution (Scytek) for 5 min, AVIDIN/Biotin blocks (VECTOR) for 20 min each at room temperature (RT) followed by a 5 min PBS wash. Biotinylated p5+14 or CGGY-p5G (control) at 1.6 μg/mL in PBS was added and incubated overnight at 4°C. Following a PBS wash Alexa Fluor 594-conjugated streptavidin (Molecular Probes) was added at a 1:200 dilution in PBS for 1h at RT. Cells were then permeabilized with 0.2% Triton X-100 (Sigma) in PBS for 10 min at RT and washed with a solution of 1% BSA in PBS for 30 min. The cells were then stained with Alexa Fluor 488-conjugated phalloidin (Molecular Probes) at a 1:100 dilution of stock in 1% BSA/PBS, for 45 min at RT to visualize actin filaments. Slides were cover-slipped using a fluorescent mounting medium (Dako) to minimize photobleaching.

### Measuring bound peptide

MEF 10.1 Cells were grown in 24-well cell culture plates as described above. Each well was probed with 100 μL of biotinylated peptides at 1 μg/mL in cold DMEM/F12 with 0.1% BSA and incubated for one hour at 4°C. Following the incubation, cells were washed twice with ice cold PBS and fixed with 1.25% glutaraldehyde. Fixed samples were washed twice and stored in PBS for 24 hours. The samples were then blocked with 1% BSA in PBS and probed with 100 μL of Europium-conjugated streptavidin (Perkin-Elmer, Waltham, MA) in PBS/0.1% BSA for 30 min at RT. The plate was washed three times with PBS and enhancement solution added. The fluorescence counts of the control peptide (*i*.*e*., background), P5+14 treated cells, and P5+14 treated cells with added enzymes were measured using time resolved fluorescence on the Wallac Victor 3 (Perkin-Elmer) plate reader. Background counts were subtracted from all treatments. The percent reduction in bound peptide was calculated as 100%-(enzyme treated fluorescence counts/no enzyme treated counts x 100).

### Statistical analysis

The data presented are pooled results from three or more experiments performed independently (*i*.*e*., repeats), with at least three replicates in each experiment. Error bars represent the standard deviation (SD). Statistical significance was calculated using one tailed student’s *t* test or 1 way ANOVA followed by Tukey's Multiple Comparison Test in GraphPad Prism following the recommendations of Vaux et al [35, 36]. Significance was determined for each separate run for each of the repeats. A *p* value < 0.05 was considered statistically significant, * = p<0.05, ** = p<0.01, *** = p<0.001, NS = non-significant reduction in infection. In the case of experiments with only three samples, statistical significance should be interpreted with caution. The small sample size could be susceptible to type II error.

## Results

### Screening of peptides

**Fig 1:**
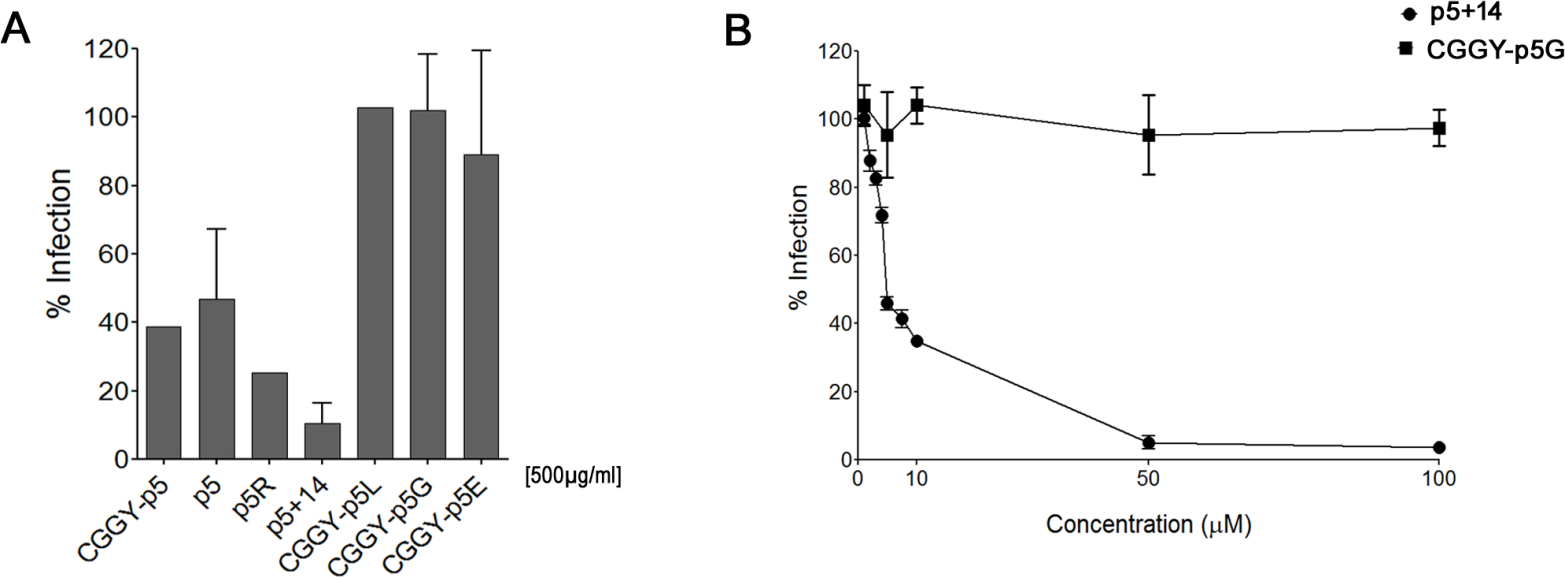
Heparin-reactive peptides reduce MCMV infection *in vitro*. (A) Peptides (500 μg/ml) with different net charges and lengths were incubated with cells 30 min prior to addition of MCMV (~100 pfu/well). Bars represent the average of the percent reduction in infection compared to PBS-treated control from three independent experiments with at least three replicates in each + SD. (B) p5+14 and CGGY-p5G (control peptide) were serially diluted and assayed in a plaque reduction assay as described in materials and methods.

Seven synthetic peptides based on the structure of peptide p5 were screened for their ability to reduce MCMV infection *in vitro* (Table 1). In the initial screening assays all peptides were tested at a single concentration (500 μg/ml) using a plaque-reduction assay, in which mouse embryonic fibroblasts were incubated with the peptides for 30 min prior to the addition of virus. The polybasic peptides exhibited a range of viral inhibition up to >90% inhibition for peptide p5+14 (Fig 1A). In contrast, the poly anionic, uncharged, and hydrophobic p5 variant peptides, CGGY-p5E, CGGY-p5G, and CGGY-p5L, respectively, did not reduce MCMV infection (Fig 1A). The presence of an N-terminal Cys residue, which was originally generated to facilitate incorporation of the radionuclide ^99m^Tc in peptide CGGYp5, did not alter the efficacy of GGGY N-terminal variant (p5) (Fig 1A). However, the CGGYp5 was prone to self-aggregation (data not shown) and was therefore not further considered in this study.

**Table 1:** Characteristics of peptides.

Peptide	Sequence	Net Charge	Plaque Reduction (Average from Fig 1)
CGGY-p5	CGGYS KAQKA QAKQA KQAQK AQKAQ AKQAK Q	+8	~61%
CGGY-p5E	CGGYS EAQEA QAEQA EQAQE AQEAQ AEQAE Q	-8	0
CGGY-p5L	CGGYS LAQLA QALQA LQAQL AQLAQ ALQAL Q	0	0
CGGY-p5G	CGGYS GAQGA QAGQA GQAQG AQGAQ AGQAG Q	0	0
p5	GGGYS KAQKA QAKQA KQAQK AQKAQ AKQAK Q	+8	~53%
p5R	GGGYS RAQRA QARQA RQAQR AQRAQ ARQAR Q	+8	~75%
p5+14	GGGYS KAQKA QAKQA KQAQK AQKAQ AKQAK QAQKA QKAQA KQAKQ	+12	~90%
G2	MPRRR RIRRR QK	+8	

Following the initial screen, peptide p5+14 was selected for further analysis because it induced the greatest reduction in infection. Serial dilution of p5+14 peptide resulted in significant reduction in infection at concentrations > 5 μg/mL (Fig 1B) with an IC_50_ of 5.2 μM.

### Structural aspects and insights into the mechanism of action

**Fig 2:**
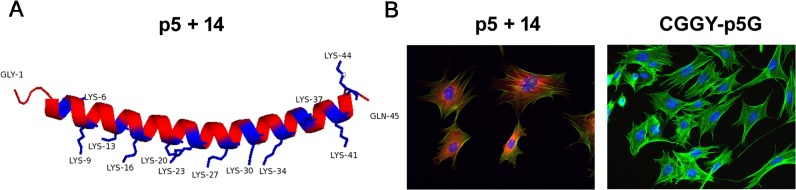
p5+14 binding to cells is charge dependent. (A) Predicted α-helix structure of peptide p5+14 based on ITASSER modeling. (B) Biotinylated peptide p5+14 (left panel) or CGGY-p5G (right panel) was added to MEF 10.1 cells followed by addition of Alexa Fluor 594-conjugated streptavidin (red). Nuclei are stained blue with Hoechst and F-actin stained green with Alexa Fluor 488-conjugated phalloidin.

ITASSER software [37, 38] predicted the secondary structure of peptide p5+14 to be α-helical with the majority of the Lys residues aligned along one face of the peptide due to the heptad repeat in the protein sequence [39] (Fig 2A).

To test our hypothesis that peptide p5+14 prevents MCMV infection by competing effectively for negatively charged cell surface HSPG, biotinylated p5+14 was incubated with fibroblasts in culture. Biotinylated peptide CGGY-p5G, which replaces Lys with Gly throughout the peptide, served as a negative control. The p5+14 bound mouse fibroblasts in culture as evidenced by the red (Alexa 540) fluorescence stain associated with the cells (Fig 2B left). In contrast, the electro-neutral peptide CGGY-p5G did not bind (Fig 2B right), suggesting that the binding of the peptide to fibroblasts was dependent upon the presence of basic (Lys) residues.

### Peptide-mediated reduction of MCMV infection through cell surface HS binding

**Fig 3:**
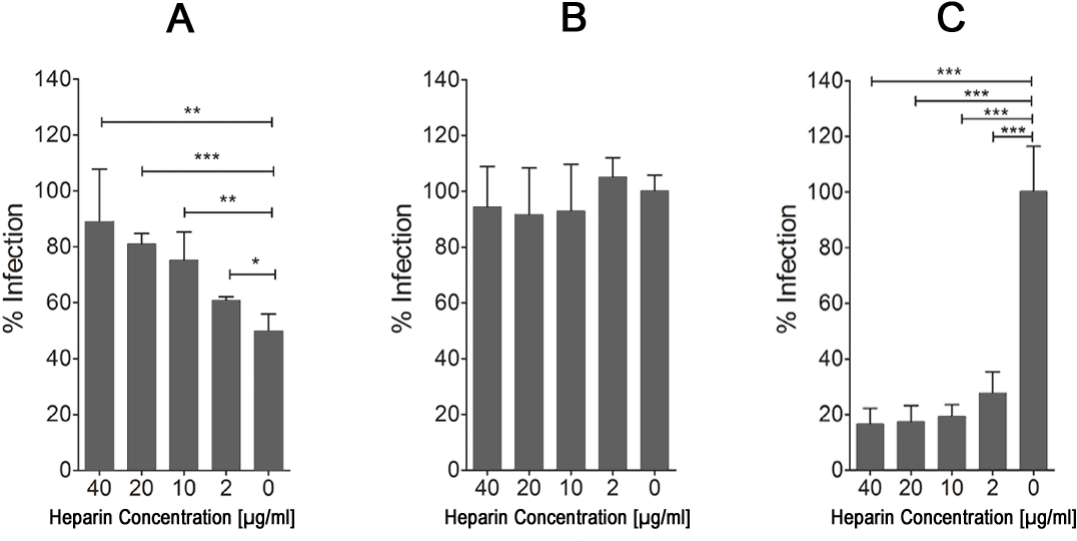
Soluble heparin interferes with peptide inhibition of virus infection. The effect of heparin on the activity of peptide and MCMV viral infectivity *in vitro* when (A) pre-incubated with the peptide (100 μM), (B) incubated with the cells before adding virus, and (C) pre-incubated with virus alone in a plaque reduction assay. Bars represent the average of the percent reduction in infection compared to PBS-treated control from three independent experiments with at least three replicates in each + SD. Statistical significance is indicated as: * = p<0.05, ** = p<0.01, *** = p<0.001.

If p5+14 binds to negatively charged HS moieties on the cell surface, pre-incubation of the peptide with heparin, which has similar charge and structural properties to HS, should interfere with peptide-mediated reduction of infection. To test this, we incubated p5+14 with various concentrations of heparin. Pre-incubation of peptide with heparin before addition to the cells reduced its ability to inhibit MCMV infection in a dose-dependent manner (Fig 3A). It should be noted that the ~50% reduction in infection with peptide and no heparin (*i*.*e*., 0ug/ml heparin concentration in Fig 3A) is different than the ~90% reduction in Fig 1. We ruled out degradation of the peptide during the pre-incubation step as an explanation for this discrepancy (data not shown). This disparity could however be due to the additional wash step after incubation of peptide + heparin. This additional wash could remove cell-surface bound peptide decreasing peptide interference with infection. This step is necessary to avoid any free heparin neutralizing the virus so it could not be eliminated from the protocol. In contrast, pre-incubation of the cells with negatively charged heparin prior to virus addition did not alter MCMV infection (Fig 3B). However, when heparin was pre-incubated with MCMV (without peptide), infection was reduced > 80% at all heparin concentrations ≥ 2 μg/mL (Fig 3C). This data supports our hypothesis that p5 is binding the negatively charged GAGs on the cell surface which can be counteracted by incubation with the negatively charged heparin [40].

**Fig 4:**
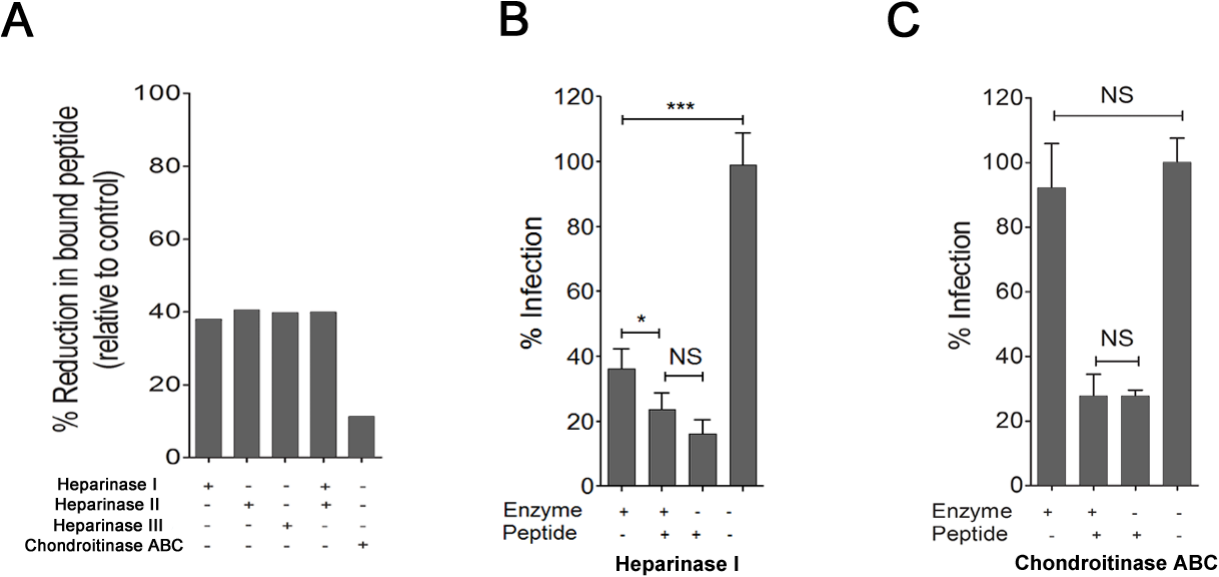
Peptide interacts with cell surface heparan sulfate but not chondroitin sulfate to mediate anti-viral activities. (A) MEF 10.1 cells were treated with heparinase I, II, III or chondroitinase ABC and the amount of bound peptide was assessed as described in materials and methods. (B) Cells were treated with heparinase I (1U/ml) or (C) chondroitinase ABC (1U/ml) and peptide p5+14 was added. The amount of plaque reduction of MCMV infection in each treatment was measured in a plaque reduction assay. Bars represent the average of the percent reduction in infection compared to PBS-treated control from three independent experiments with at least three replicates in each + SD. Statistical significance is indicated as: * = p<0.05, ** = p<0.01, *** = p<0.001, NS = non-significant difference in the reduction of infection.

Because p5+14 can bind both HS and CS GAGs, the reduction in infection could be mediated by direct competition for virus adsorption sites via HS on the cell surface, stearic hindrance mediated by peptide bound to CS on the cell surface, or both. To distinguish between these possibilities, cells were treated with heparinase or chondroitinase enzymes to cleave the different GAGs from the cell surface. Treatment of cells with heparinase caused a ~40% reduction in the amount of bound peptide, whereas chondroitinase treatment resulted in a ~11% reduction (Fig 4A). Treatment of cells with heparinase (1U/ml) led to a ~60% reduction in MCMV infectivity as expected, which was enhanced further by the addition of p5+14 leading to an ~80% reduction (Fig 4B). There was no significant difference between peptide alone and peptide in conjunction with heparinase treatment. In contrast, treatment of cells with chondroitinase (1U/ml) did not reduce MCMV infectivity nor did it have any effect on the activity of the peptide (Fig 4C). Pre-treatment of MCMV itself with heparinase or chondroitinase prior to addition to MEFs did not alter its infectivity (data not shown). These results indicate that p5+14 blocks MCMV infectivity via heparan sulfate and not steric hindrance after binding to chondroitin sulfate.

### Peptide competes for virus adsorption to the cell surface

**Fig 5:**
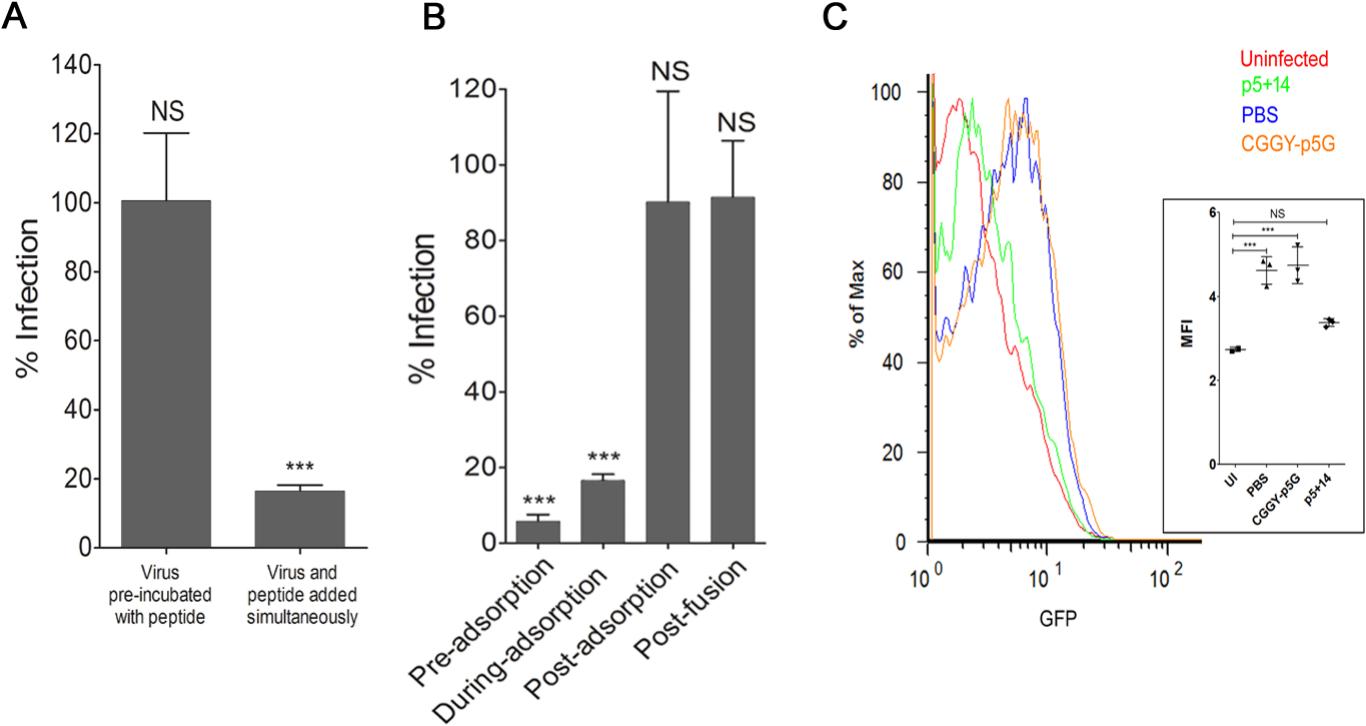
Peptide p5+14 blocks adsorption of MCMV. (A) MCMV was preincubated with p5+14 peptide (100 μM) before diluting the virus/peptide to an ineffective peptide concentration (1 μM) and assayed in the plaque reduction assay as described in materials and methods. As a control, virus and peptide (100 μM) were added to cells simultaneously. (B) Cells were incubated with p5+14 peptide either prior to virus adsorption, during virus adsorption, after virus adsorption (at 4°C) but prior to fusion, or after fusion (at 37°C). Plaque reduction was measured in a plaque reduction assay. Bars represent the average of the percent reduction in infection compared to PBS-treated controls from three independent experiments with at least three replicates in each + SD. Statistical significance is indicated as: * = p<0.05, ** = p<0.01, *** = p<0.001, NS = non-significant difference in the reduction of infection compared to PBS treated control wells. (C) Adsorption of HCMV TB40/E-pp150-GFP (MOI 10) fusion protein expressing HCMV was measured via flowcytometry in the presence of p5+14 (green), control peptide CGGY-p5G (orange) and PBS (blue). Red line represents uninfected cells. Inset is a scatter plot of the mean fluorescence intensity (MFI) for GFP with the line representing average of 3 replicates +/- SD for the different treatments.

In the infectivity assays described above, peptide and virus were co-incubated with the cells. In this experimental setup, the peptide could bind to the virus, to cells, or both and reduce infection. Therefore to ensure that the peptide was not directly inactivating the virus, MCMV was co-incubated with 100μM (~20x the IC_50_) peptide at 37°C for 1 h, diluted to an *ineffective* peptide concentration (1 μM) and infection of fibroblasts measured. There was no reduction in MCMV infection under these conditions, whereas addition of peptide and virus simultaneously to the cells showed significant reduction in infection (Fig 5A).

To determine at which stage of the MCMV entry cycle the peptide interferes, four different peptide treatment protocols were tested: 1) 30 min prior to infection (pre-adsorption) 2) simultaneously with virus (during adsorption) 3) after letting the virus adsorb to the cells at 4°C for 1h (post adsorption, then shifted to 37°C to induce membrane fusion) or 4) after allowing the virus to fuse with the cellular membrane at 37°C for 1h (post fusion) (Fig 5B). Addition of the p5+14 peptide before or in conjunction with virus addition to the cells resulted in >80% reduction in infection. However, when peptide was added after the adsorption or fusion phase of viral entry, no significant reduction in plaque formation was observed (Fig 5B).

To specifically show that p5+14 prevents adsorption of HCMV to cells, HCMV expressing a tegument protein-green fluorescent fusion protein, pp150-GFP, was incubated with HFF cells at 4°C in the presence or absence of the different peptides. The fluorescence of cell-associated virus was measured via flowcytometry (Fig 5C). Incubation of the cells with p5+14 reduced the amount of fluorescent virus attached to the cell surface, whereas there was no reduction in fluorescence when cells were incubated with peptide CGGY-p5G compared to PBS treated cells.

### Comparison of p5+14 to other inhibitory peptides

**Fig 6:**
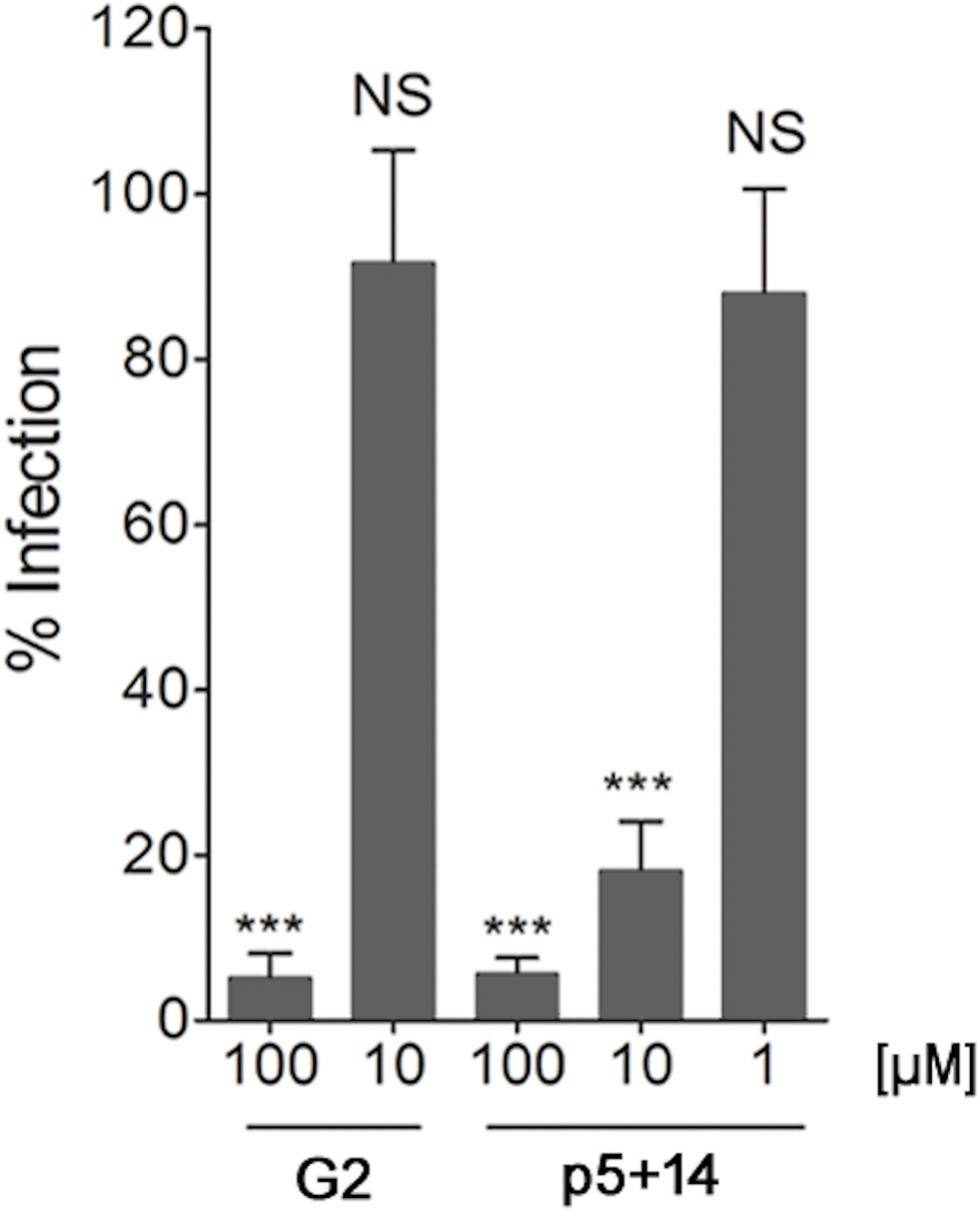
Comparison of the efficacy of p5+14 and peptide G2 to reduce MCMV infection in vitro. Peptides G2 and p5+14 were added at different concentrations (100, 10, 1 μM) in a plaque reduction assay as described in materials and methods. Bars represent the average of the percent reduction in infection compared to PBS-treated control from three independent experiments with at least three replicates in each + SD. Statistical significance is indicated as: * = p<0.05, ** = p<0.01, *** = p<0.001, NS = non-significant difference in the reduction of infection compared to PBS-treated control wells.

The efficacy of p5+14 to reduce infection was compared to the recently reported inhibitor peptide, G2, which was also inhibits infection of herpes viruses (HSV and MCMV) [41] (Table 1). Both peptides effectively inhibited MCMV infection at 100 μM, but p5+14 leads to a >80% reduction in infection at 10 μM at which concentration peptide G2 was ineffective (Fig 6).

### p5+14 inhibition of other herpesvirus infection

**Fig 7:**
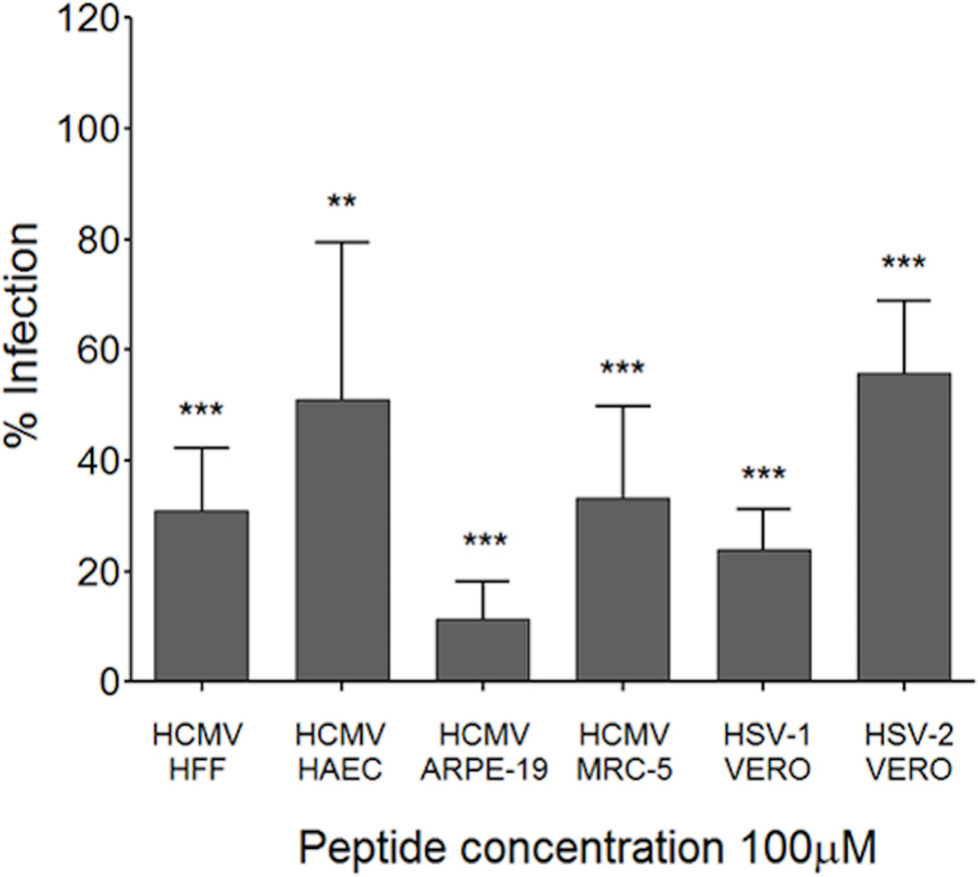
Peptide p5+14 inhibits HCMV and HSV infections *in vitro*. Peptide p5+14 (100 μM) was added in a plaque reduction assay using HCMV (TB40/E) on different cell types (HFF, HAEC, and ARPE-19) and HSV-1 or HSV-2 on VERO cells. Bars represent the average of the percent reduction in infection compared to PBS-treated control from three independent experiments with at least three replicates in each + SD. Statistical significance is indicated as: * = p<0.05, ** = p<0.01, *** = p<0.001.

Because most herpesviruses use HS for their initial attachment to cells and can infect different cell types, we evaluated the efficacy of the peptide to block infection of other human herpesviruses infecting different cells types. Addition of peptide p5+14 at a concentration of 100 μM 30 min prior HCMV infection resulted in a reduction of ~70% on HFF, ~50% on HAEC, ~90% on ARPE-19 and ~ 60% on MRC-5 cells (Fig 7). An ~80% reduction in infection was observed with herpes simplex virus (HSV) 1. However, reduction of HSV 2 infection was less remarkable (~40% reduction).

## Discussion

Cytomegalovirus infection is a significant clinical problem in infants and immunodeficient populations. There are two major problems with current anti-CMV treatments. First, current anti-CMV therapies have significant organ toxicity. Secondly, resistance to current therapies is increasing. In this study we examined a panel of synthetic peptides that bind hypersulfated GAGs for their ability to inhibit herpesvirus infection, using MCMV as a model system. Of the seven peptides evaluated in this study, peptide p5+14 demonstrated effective inhibition of MCMV infection and reduced infection of both HCMV and HSV (HSV-1 and 2) *in vitro* (Figs 1A and 7). This suggests a broader applicability of GAG-binding synthetic peptides for inhibiting virus-cell interactions. We established that the peptide effectively competed for adsorption of CMV to susceptible cells, thereby reducing infection. We also demonstrated that the peptide does not have a direct neutralizing effect on the virus itself.

The p5-related peptides are synthetic polybasic reagents with a predicted α-helical secondary structure. The heptad amino acid repeat-KAQKAQA- positions the Lys residues along one face of the helix. This structural feature was engineered and intended to facilitate an interaction with linear sulfated GAG molecules, notably heparin [39, 42]. Due to their ability to preferentially bind hypersulfated GAGs these peptides have been used to effectively target and image tissue amyloid deposits [25, 27], which contain hypersulfated HS and possibly CS proteoglycans [43]. Remarkably, when radiolabeled the p5 and p5+14 peptides were injected in disease-free mice, peptide did not bind to GAGs expressed in healthy organs or tissues [25]. This lead us to hypothesize that the linear positive charge on peptide p5+14 facilitates binding to negatively charged PGs on the cell surface, which mediates antiviral activity. This is supported by the fact that peptides with the same net positive charge exhibit differential anti-viral effects that are consistent with the peptide affinity for the GAGs and subsequently amyloid. Thus, peptide p5R (+8 charge), which has a higher affinity for heparin [39] and amyloid [44] as compared to peptide p5 (+8 charge), blocks viral infection 2-fold better (Fig 1A). These data suggest that the secondary structure of the peptides, as well as the overall net charge, affects binding to specific GAGs on the cell surface and their subsequent anti-viral activity. Based on the known restricted reactivity of peptides p5 [25] and p5+14 *in vivo*, our data using p5+14 suggests that CMV may preferentially bind hypersulfated GAGs, such as 6-O-sulfated GAGs [40] on the cell surface of cultured fibroblasts. This may differ from the ubiquitously expressed GAGs found in tissue HSPG and CSPG proteins *in vivo*. This is similar to the proposed mechanism for HSV that uses multiple different interactions for entry including 3-O sulfated GAGs, which differ between cells grown *in vitro* and *in vivo* [45].

An alternative mode of action for these peptides may involve internalization of the peptide along with the GAG ligands that the virus uses for entry. For example, peptides that are rich in Arg or Lys are known to bind HS on the cell surface resulting in internalization of the peptide/HS complex [46, 47]. Because of this mechanism, these peptides are being considered for drug delivery or diagnostic/therapeutic nanoparticles [48–50]. It remains to be evaluated whether peptide p5+14 binding to the HSPG ligands results in internalization of the peptide-ligand complex, resulting in less HS for virus to bind and enter. These studies are underway. If this is the case, it would provide an alternative explanation for HS binding peptides’ inhibition of CMV infection and suggest that p5+14 could also be used as a reagent for delivery of intracellularly active payloads.

Tiwari *et al*. [41] and Borst *et al*.*’s* [40] recent work identified HS-reactive anti-viral peptides G2 and CYVIP from a phage library screen and human hemofiltrate, respectively. In concordance with our findings, the positive charge of these peptides was critical for their anti-viral activity. Notably, our peptide p5+14 was more effective at lower concentrations in inhibiting MCMV infection of mouse fibroblasts *in vitro* when compared with peptide G2 (Fig 6). Although these peptides have similar modes of action, there are significant differences in their size and charge distribution. The length and spatial arrangement of charged amino acids affect binding to heparin [39], HS-laden amyloid [51], and cell surface HS [46]. Although we used the L form of the peptide G2 in the current study, recently the D form of the G2 peptide was shown to be 4 times as efficacious as the L form *in vitro* [52]. This form has the additional advantage of being proteolytically stable. Thus the authors propose that D form could be important for *in vivo* treatments because it would be more stable in serum. A systematic evaluation of the physical, electrochemical, and structural characteristics that contribute to anti-viral activity of all these peptides will aid in the design of next generation antivirals.

In this study we show that peptide p5+14 exhibited significant anti-viral activity against HCMV, HSV-1 and 2. It is interesting that the antiviral effects were more robust on the HSV-1 than on HSV-2. Even though we propose a similar mode of action against each virus (*i*.*e*., blocking of viral adsorption to cell surface HS) the difference in peptide p5+14 efficacies is intriguing. Differences in the viral gB glycoproteins could lead to preferential use of specific GAGs to adsorb to the cell surface [53] that lead to differences in the efficacy of the peptide against the two HSV serotypes. Indeed, the fine structure and distribution of HS GAGs can be different on different cell types. This can explain differences in the efficiency of peptide blockade on different strains and cell types [54, 55]. It is possible, indeed likely, that the p5+14 peptide and similar reagents exhibit preferential binding to GAGs that could lead to differences in cell-surface binding and antiviral efficacy. Notably, circular dichroism measurements showed that peptide p5 preferentially binds heparin and adopts an α-helical configuration compared to HS, CS, dermatan sulfate, and hyaluronic acid [42].

Using SPECT imaging and micro-autoradiography, we have previously shown that the “ligand” bound by peptides p5 [25] and p5+14 (unpublished data) has a restricted distribution *in vivo*. The peptides do not bind cellular GAGs or those in the extra-cellular matrix of healthy tissues [25]. This observation, taken together with the fact that these peptides compete with herpesviruses for binding to cells in culture suggests that viruses may preferentially bind to a subset of HS *in vivo* that is characterized by a high sulfation pattern, (*i*.*e*., electrochemically more reminiscent of heparin). This pattern has been observed with HSV [22, 53]. This remains to be established *in vivo*.

CMV and other herpes viruses establish latency within the host, which is dependent upon virus entry and infection of host cells. Preventing viral entry using competitive peptides could potentially reduce the ability of virus to establish latency. Even though HS on the cell surface is an attractive target for developing antivirals, reports targeting this pathway during viral infections *in vivo* are scarce [41]. This is likely due to the fact that HS is ubiquitous and involved in numerous critical cell-signaling pathways. Thus, peptides such as p5+14 that specifically targeted heparin-like HS may provide selective viral competition *in vivo* without detrimentally affecting biological processes through a more common HS.
